# A Rare Case of Congenital Intestinal Malrotation Presented as an Intestinal Obstruction in an Adult

**DOI:** 10.7759/cureus.49812

**Published:** 2023-12-01

**Authors:** Fathi Elgeyoushy, Amal Sayfuldeen Qari, Raneem Ahmed Faidh

**Affiliations:** 1 General Surgery, King Fahad General Hospital, Medina, SAU; 2 College of Medicine, Taibah University, Medina, SAU

**Keywords:** intestinal obstruction, adult intestinal malrotation, ladd’s bands, midgut malrotation, abdominal pain

## Abstract

Malrotation of the midgut is generally considered a pediatric pathology. Adults are not usually diagnosed early, which occasionally causes delays in both diagnosis and treatment. Therefore, while dealing with patients of any chronological age with abdominal complaints, a strong index of suspicion is necessary. A midgut developmental defect known as intestinal malrotation occurs when the intestines fail to fix in the peritoneal cavity and rotate normally around the superior mesenteric artery during fetal development. Usually, it’s rare to have malrotation in adults. When they are symptomatic, operational procedures are typically necessary. Even if the patient is asymptomatic, a Ladd's treatment is indicated if real malrotation is detected or discovered accidentally. In adults, intestinal malrotation rarely shows symptoms and is typically accidentally discovered. We present a unique case of a 24-year-old male who had acute abdominal pain, confirming midgut rotation with the presence of characteristic Ladd's bands on a preoperative computed tomography scan. No signs of intestinal volvulus were present. The patient had an exploratory laparotomy. This case emphasizes the rarity of intestinal malrotation and the debates about how to treat it in the adult population. It also highlights how crucial it is to properly monitor patients who present with ill-defined abdominal pain and maintain a high index of suspicion.

## Introduction

Intestinal malrotation is a congenital anomaly that causes the duodenojejunal junction to be positioned in the right middle during development by rotating the gut around the superior mesenteric artery axis. Although it has early-onset symptoms, a few individuals (0.1-0.5%) live until adulthood with no symptoms [1]. The prevalence of cases reported is one in 6000 and one in 200 of all live births, with most cases occuring in the first month of life and 90% within the first year. Due to the vague nature of malrotation in adolescence and adulthood, the radiologist may be the first to arrive at this crucial diagnosis because of an accidental imaging discovery or as the reason for unexpected abdominal symptoms [1,2]. Several congenital obstructive defects, such as duodenal atresia and stenosis, are related to intestinal malrotation. However, there have only been a few cases of intestinal blockage brought on by a duodenal web in this setting of malrotation. Both these pathologic entities can produce poorly defined symptoms like nausea and abdominal pain. Delays in diagnosis and treatment might result in higher mortality and morbidity [3]. Our case report highlights the importance of considering intestinal malrotation as a potential diagnosis in adult patients presenting with gastrointestinal symptoms; it is about a 24-year-old male with a clinical picture of intestinal obstruction due to congenital intestinal malrotation.

## Case presentation

A 24-year-old male heavy smoker who had a history of congenital diaphragmatic hernia, which was repaired in infancy, presented to the Emergency Department (ED) with acute abdominal pain for seven days associated with constipation and vomiting. The pain was sharply localized in the epigastric region, with no radiation or association with food. The pain was rated 9/10. He denied any recent falls, injury, or exposure to ill contact. The patient did not have fever, dysuria, or perineal hemorrhage. The results of the systematic review were insignificant, neither a notable family history nor regular drug use.

**Figure 1:**
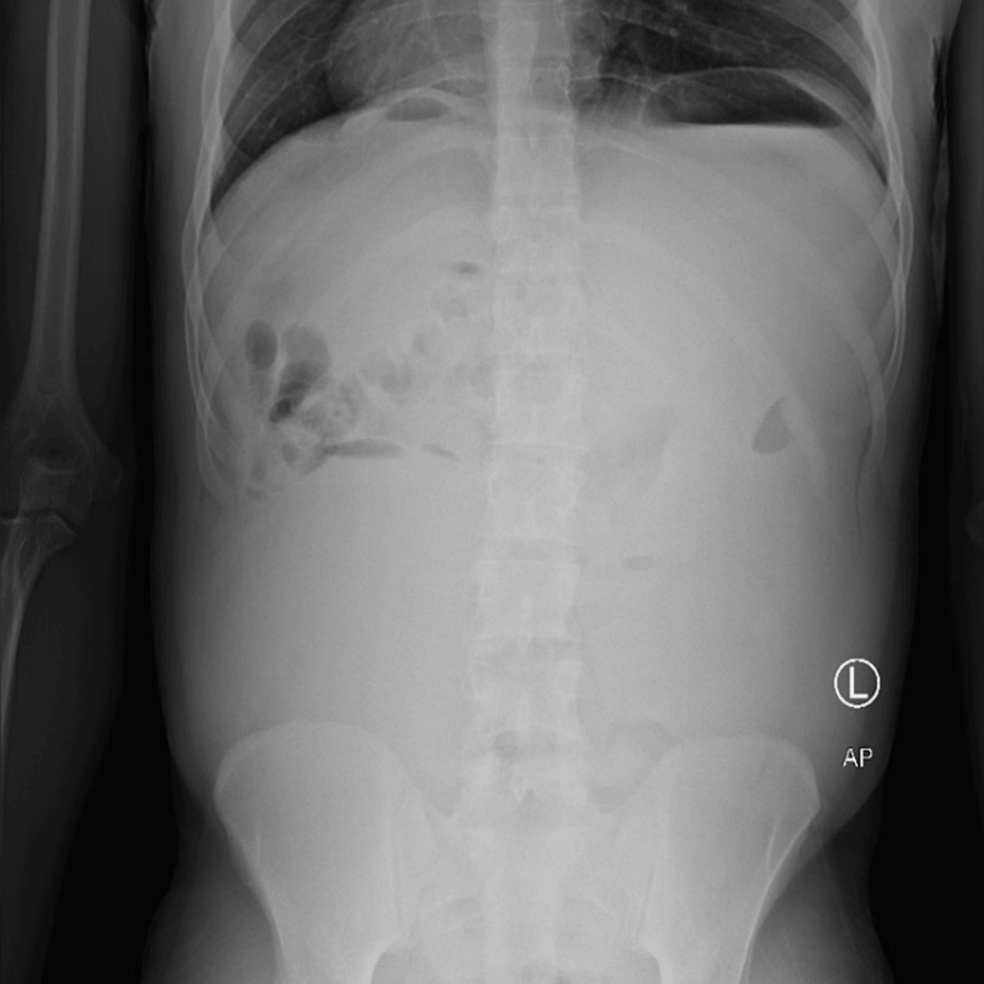
Chest and abdominal x-ray

Upon physical examination, his vital signs were normal except for a tachycardia at 120. Along with rebound tenderness and voluntary guarding, the abdomen was diffusely tender. He had a slight distension of the abdomen. Additional systemic examination revealed nothing noteworthy. His first lactate level upon presentation was 4.6 mmol/L, which increased to 8.6 mmol/L when tested again two hours later. His blood tests were otherwise rather unremarkable. An x-ray of the chest and abdomen was taken, and the results showed no abnormal conditions, such as dilated bowel loops or free air under the diaphragm (Figure 1).

**Figure 2:**
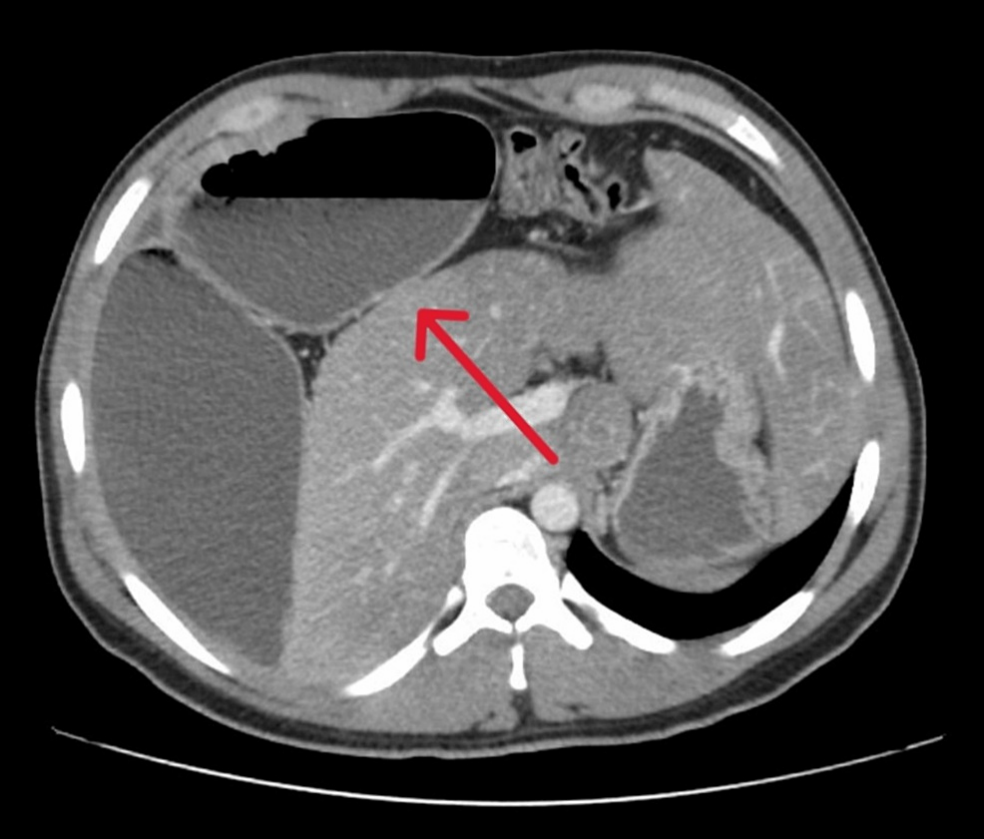
Dilated small bowel (jejunum located in right subphrenic space between the right copula of diaphragm and right lobe of the liver)

The patient was resuscitated with fluids and prepared for pelvic and abdominal CT which showed dilated small bowel loops mainly at the jejunum reaching approximately 10 cm in maximum diameter in the right upper quadrant with air-fluid level seen. There was no pneumoperitoneum and no pneumatosis intestinalis. Severe hepatic steatosis was noted. However, there was no obvious focal lesions and no signs of ischemia or perforation (Figure 2).

Following CT, the patient was prepared for exploratory laparotomy. Finding largely distended small bowel loops (proximal jejunal), diameter 10 cm with intact bowel walls no signs of ischemia or perforation. Ladd's band was noted extending on the right side compressing the second part of the duodenum and extending upwards. The cecum was noted adherent to the duodenum with the band, large bowels are noted to be collapsed with an intact wall and no signs of ischemia. Transition zones were noted at the jejunoileal junction, due to the adhesive band from the previous left thoracotomy.

Examination of the bowel started from the duodenum with the release of the Ladd's band done till the compression at the third part of the duodenum was released; an examination of large and small bowel was then done with the findings mentioned above noted, and extensive adhesiolysis done to release the bowel lobe from the adhesive bands. At the previous left thoracotomy scar, dense adhesive bands were noted between the abdominal wall, small bowel loops, and the spleen along with the left lateral loop of the liver, all of which were released using a sharp and ligasure device.

Upon release, a 1 cm capsular spleen injury occurred which was controlled intra-op, and no significant bleeding was noted from the spleen. Adhesiolysis continued till all the small bowel loops were released. Afterward, a 5 cm mesenteric defect was noted, closed continuously using a PDS3/0 suture. A prophylactic appendectomy was done due to abnormal anatomical position of the appendix. Then the large bowel was placed on the left abdominal cavity and small bowel loops were reduced on the right side of the abdominal cavity.

A nasogastric tube (NGT) was inserted and then the distended jejunal loop contents were milked towards the stomach to decompress it, and around 1000 ml of greenish fluid was suctioned. The abdominal cavity was washed with warm normal saline and a drain was inserted. Laparotomy was closed. Histopathology of the appendix showed mild acute appendicitis.

He had an uneventful postoperative recovery and was discharged home two weeks post surgery. The patient did well, tolerating oral food, passing motion, and ambulating. He was vitally stable and afebrile, and his abdomen was soft and lax, with no tenderness or distension. Clips were removed on postoperative day 10, and OPD follow-up was after two weeks.

After one month of OPD follow-up, he complained of abdominal discharge and generalized abdominal pain for one day Abdominal and pelvis CT with contrast showed loculated fluid collection and peritonitis, newly developed enterocutaneous fistula due to perforation secondary to ischemia of small bowel loop.

**Figure 3:**
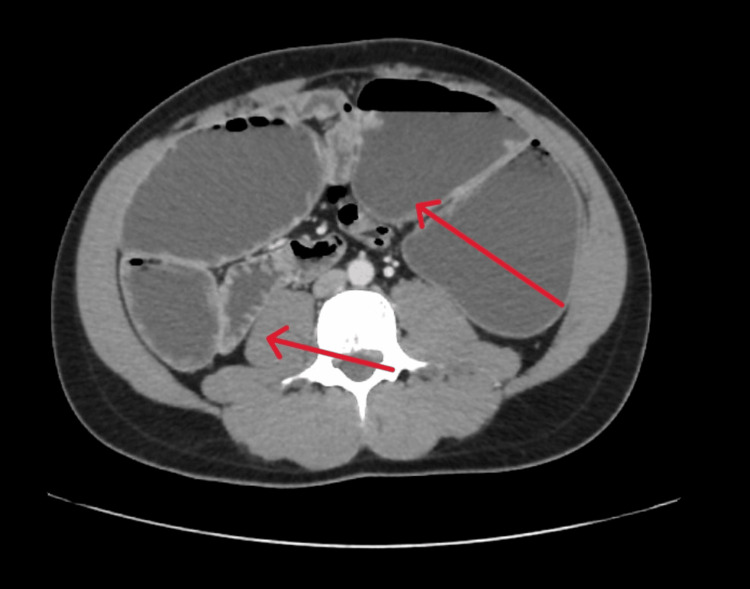
Transition zone at mid-abdomen at the level of the loop of mesentery associated with twisting of mesenteric vessels.

In the second laparotomy, the abdomen was examined with a dilated proximal small bowel, perforation in the loop of the small loop (jejunum), which was ischemic and the small loop was resected and jujenostomy. The dilatation of small bowel loops starts with gradual transitional zone from the second/third part of the duodenum ending gradually with collapsed distal bowel loops at the midbowel at the level of small bowel loops are abnormally situated on the right side of the abdomen, while large bowel loops observed on the left. The mesentery root is twisted, and the mesenteric vessels affect on liver with displacement toward the midline (Figure 3).

**Figure 4:**
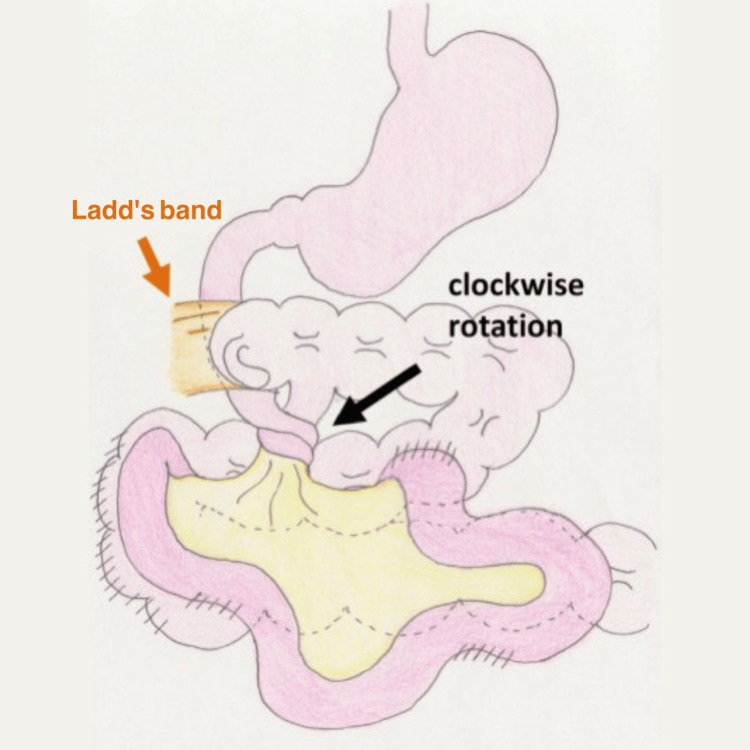
Surgical findings. The ileocecum presented at the left side of abdomen, and the Ladd ligament was present crossing the second part of the duodenum and attached to posterior abdominal wall lateral to duodenum Image Source: Higashi et al., 2021 [4]; open access under the CC BY-NC-ND license (http://creativecommons.org/licenses/by-nc-nd/4.0/).

Figure 4 was taken from Higashi et al.'s article that shows the surgical procedure [4]. The ileocecum presented at the center of the upper abdomen, and the Ladd ligament was present. The intestine was highly adherent.

**Figure 5:**
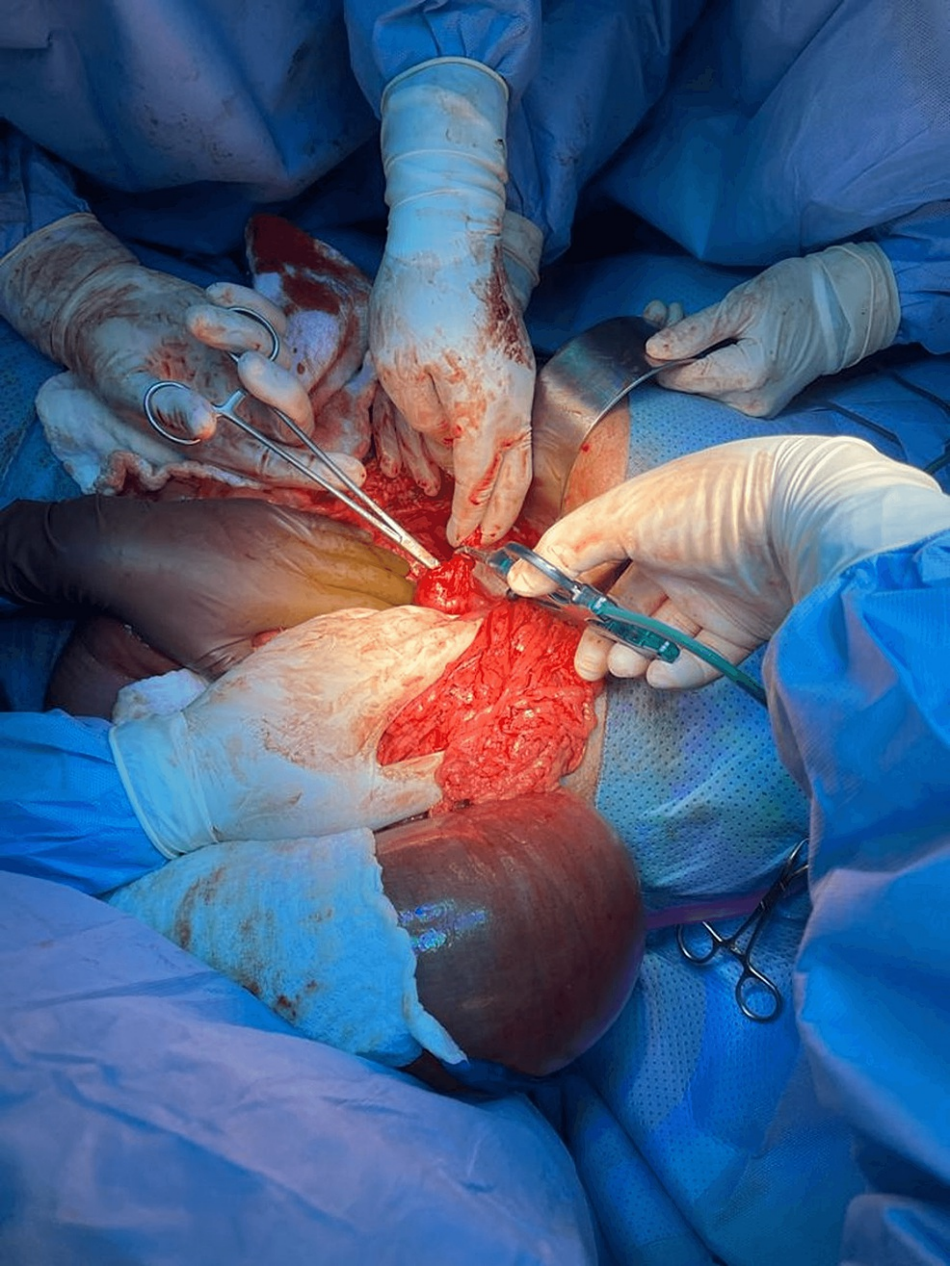
Exploratory laparotomy showed division of the band

After one week, he went for a third exploratory laparotomy due to subhepatic collection of abscess drainage (Figure 5).

After three months, the patient came back for closure of jejunostomy and restoration of bowel continuity and tolerated the procedure well. He has been followed in OPD and was doing fine gaining weight and in good health condition.

## Discussion

Ninety percent of intestinal malrotation cases are discovered within the initial year of life, leading to a congenital defect. With a 0.2% estimated prevalence, adult presentation is uncommon. Many cases are discovered by chance during imaging or surgery for other comorbidities [5]. The clinical diagnosis is usually not considered in the first evaluation due to the vague presentation and progressively decreasing index of suspicion for malrotation in the adult population [6].

The 270° counterclockwise gut tube's rotation around the superior mesenteric artery (SMA) axis is required for appropriate midgut development. The retroperitoneal attachments to the duodenum and colon, the ileocecal valve in the right lower quadrant, and the ligament of Treitz in the left upper quadrant are formed by this rotation, which typically takes place between the fourth and tenth weeks of gestation. In the typical anatomical structure, volvulus is prevented by this broad-based mesentery [7].

The inability of the midgut's natural physiological rotation to occur results in varying degrees of abnormality; it's also possible for the peritoneal fibrous bands to continue connecting the duodenum and caecum to the abdominal wall, and the small bowel mesentery may also form a limited vertical attachment. These congenital bands, also referred to as Ladd's bands, originate from the abdomen's posterior wall, crossing the duodenum and attached to the undescended caecum. The duodenum is compressed by Ladd's bands, which may result in duodenal obstruction. The anomalous position of the caecum and the malrotation of the gut result in a narrow superior mesenteric vascular pedicle, in contrast to the normally broad-based small bowel mesentery. This incomplete posterior peritoneal fusion and restricted SMA takeoff increase the risk of midgut volvulus and obstruction, which could have catastrophic vascular effects [8,9].

Acute clinical manifestation of malrotation is manifested by signs of peritonitis, small intestinal obstruction, or appendicitis. Due to disturbance of the appendix's normal location that may occur from malrotation abnormalities, signs may not be in the right lower quadrant. The abnormal anatomy could make it challenging to see the presentation of other abdominal problems [5,10].

With increased and extensive use of radiographic studies, we can anticipate a rise in the incidental identification of intestinal malrotation. Plain abdominal radiographs, ultrasound, CT scans, MRI, and mesenteric arteriography can all be used to diagnose midgut malrotation [11,6].

In contrast to young populations, diagnosing intestinal malrotation in adults is harder to identify. Upper GI series is the preferred diagnostic approach for children; however, previous case reports and series show that adults, particularly those who present with more distal obstruction, are not as sensitive to these tests [11,8,12].

William Ladd originally detailed the surgical care of intestinal malrotation in 1936, and this is still the cornerstone of treatment today [13]. Ladd's procedure is an operation for intestinal malrotation that includes appendectomy, volvulus detorsion, release of Ladd's bands, and expansion of the mesenteric base. Due to greater clinician comfort and a lower risk of an incomplete procedure, open surgery is typically preferred to laparoscopic surgery, especially when accessing posterior duodenal attachments [11,8].

## Conclusions

This report emphasizes the importance of considering intestinal malrotation as one of the differential diagnoses in adults with gastrointestinal symptoms. While intestinal malrotation is commonly associated with pediatric patients, it can manifest in adulthood and present with diverse clinical features, making diagnosis challenging. Early recognition of intestinal malrotation is crucial to avoid potentially serious complications, such as volvulus or intestinal ischemia. Surgical intervention remains the mainstay method of treatment for symptomatic intestinal malrotation in adults. Timely surgical correction, which typically involves Ladd's procedure, reduces pain, avoids difficulties, and enhances the patient's standard of living. Furthermore, this case report emphasizes the need for increased awareness among healthcare professionals regarding the possibility of intestinal malrotation in adults. Enhanced knowledge and vigilance will lead to earlier diagnosis and appropriate management, ultimately improving patient outcomes. Overall, this report prompts that intestinal malrotation should be considered in the differential diagnosis of adults presenting with gastrointestinal symptoms and highlights the importance of a multidisciplinary approach involving gastroenterologists, surgeons, and radiologists to ensure accurate diagnosis and optimal patient handling.
